# Physical capability after total joint arthroplasty: long-term population-based follow-up study of 6,462 women

**DOI:** 10.1080/17453674.2021.1922039

**Published:** 2021-05-12

**Authors:** Ville Turppo, Reijo Sund, Jukka Huopio, Heikki Kröger, Joonas Sirola

**Affiliations:** a Kuopio Musculoskeletal Research Unit (KMRU), Institute of Clinical Medicine, University of Eastern Finland (UEF), Kuopio; b Department of Orthopaedics, Traumatology and Hand Surgery, Kuopio University Hospital, Kuopio, Finland

## Abstract

Background and purpose — There is lack of knowledge concerning patient-reported long-time outcome after arthroplasty. Therefore, we investigated patient self-reported physical capabilities (PC) and subjective well-being (SW) up to 20 years after total hip (THA) or knee (TKA) arthroplasty.

Subjects and methods — The self-reports from postal questionnaires for study checkpoints (baseline, 10-year follow-up, 20-year follow-up) were provided by the Kuopio OSTPRE study including only women aged 52–62 years (n = 6,462). The Finnish Arthroplasty Register and Care Register for Health Care provided data on arthroplasties in the OSTPRE population. The results of women with THA/TKA were compared with women without arthroplasty (control group).

Results — In subjects with THA performed before the 10-year follow-up, the proportion of good PC was initially decreased by 0.6 percentage points (pp) at the 10-year follow-up and later by 19 pp at the 20-year follow-up. After TKA, the proportion of subjects with good PC decreased by 4.1 pp (10–year follow-up) and 27 pp (20-year follow-up), respectively. The proportion of controls reporting good PC decreased by 1.4 pp at the 10-year follow-up and 14 pp at the 20-year follow-up compared with the baseline. After THA, the proportion of subjects with good SW stayed on the same level at 10-year follow-up and decreased by 2.3 pp at 20-year follow-up. After TKA, the proportion of good SW increased by 9.0 pp (10-year follow-up) and decreased by 14 pp (20-year follow-up). The proportion of controls reporting good SW increased by 4.0 pp (10-year follow-up) and decreased by 8.8 pp (20-year follow-up).

Interpretation — THA and TKA maintain PC and SW. The overall PC and SW are lower in women with arthroplasty, in comparison with controls without arthroplasty. THA seems to outperform TKA in maintaining PC.

In recent years, more attention has focused on patient-reported outcomes after total hip (THA) and knee (TKA) arthroplasty. Most studies on patient-reported outcome measures (PROM) have relatively short follow-ups (Ethgen et al. 2004). As implants will usually survive longer, there is a need to investigate long-term patient satisfaction and functioning.

We found only a few PROM studies reporting long-term results on THA and/or TKA. THA seems to have high patient satisfaction and good functional outcomes, up to at least 16 years after operation (Mariconda et al. 2011, Gould et al. 2012). TKA seems to maintain patient functioning and activity up to 20 years postoperatively (Meding et al. 2012).

Patients often inquire about the performance of THA and TKA in activities of daily living. Also, the performance of THA and TKA is compared, by patients, with non-operated knees and hips. However, there are no studies available that have compared the physical capability and subjective well-being between THA and TKA patients and non-operated patients. Also, the long-term changes in PC and SW after THA and TKA remain largely unknown.

We assessed long-term patient self-reported physical capability (PC) and subjective well-being (SW) in women even up to 20 years after a primary THA or TKA. We compare THA/TKA patients with a control group and postoperative scores were compared with preoperative scores.

## Subjects and methods

This study is based on the long-term follow-up of the female population in the Kuopio Osteoporosis Risk Factors and Prevention study (OSTPRE). The self-reports on participants’ PC and SW were provided by OSTPRE. Supplementary data on all THAs and TKAs in the OSTPRE study population was obtained from the Finnish Arthroplasty Register (FAR) and the Care Register for Health Care (CRHC).

The original purpose of the OSTPRE study was to investigate osteoporosis in the female population in a prospective study setting. However, it has expanded from its start in 1989 into an overall health and subjective well-being cohort, still including only a female population (http://www.uef.fi/en/web/kmru/ostpre). The original study cohort included all 47–56-year-old women (n = 14,220) living in Kuopio Province in Eastern Finland in 1989. The study is based on self-reports via postal questionnaires, and it has been renewed every 5 years. In the current study, the OSTPRE 1994 questionnaire (n = 11,954) is used as baseline. Follow-ups are the 2004 (10-year follow-up, n = 10,912), and 2014 (20-year follow-up, n = 7,765) questionnaires. We chose these questionnaires to achieve long enough follow-up times for the participants. We focused on questions concerning self-reported PC and SW. These questions have basically remained the same since the questionnaire in 1994. Only those who had returned all 3 questionnaires were included in the study. The self-reported hip fractures in OSTPRE, also included in the present study, were complemented with the hip fractures found from the CRHC and all were also checked from the medical records.

The questions asked for self-reports in OSTPRE were as follows (originally in Finnish): “Describe your current physical capability?” and “How would you describe your current well-being?”. Self-reported original PC included the following answer options: 1, capable of moving without limitations; 2, no running, without other limitations; 3, can move less than 1,000 meters; 4, can move less than 100 meters independently; 5, can move only indoors; 6, I’m temporarily immobilized; 7, I’m permanently immobilized. For statistical purposes (group size), answers 1 and 2 were combined as the group “walking without limitations” and are referred later as “good PC.” Also, answers 4–7 were considered as one group, “can move less than 100 meters independently.” This classification works well in clinical settings too, since being able to walk less than 1,000 meters supports the indication for arthroplasty. Originally, SW answers formed 5 groups: very good, good, moderate, poor, and bad. Again, for statistical purposes, very good and good were combined as “good.” Poor and bad were combined as “poor.”

THA/TKA register data was collected from the FAR and CRHC. We used 2 different data sources, since it has been previously found to more comprehensively cover all arthroplasties (Turppo et al. 2018). The CRHC records all special healthcare hospital admissions. It holds records of arthroplasty operations since 1987. The FAR has recorded data from arthroplasties since 1980 (National Institute of Health and W 2019). The data was collected until 31 December 2016. Any anomalies in data were manually checked from the questionnaire forms and medical reports and corrected when possible. There were 2,444 women with THA or TKA before the final return date of the 20-year follow-up questionnaire (December 31, 2014). 921 participants who failed to return any of the

w3 questionnaires were excluded, of whom 293 had died during follow-up. 92 women underwent arthroplasty before baseline and 612 women had more than 1 operated joint. Eventually, there were 819 women with a THA or TKA who met the inclusion criteria. These women formed groups according to the time of their THA or TKA.

**Figure 1.**
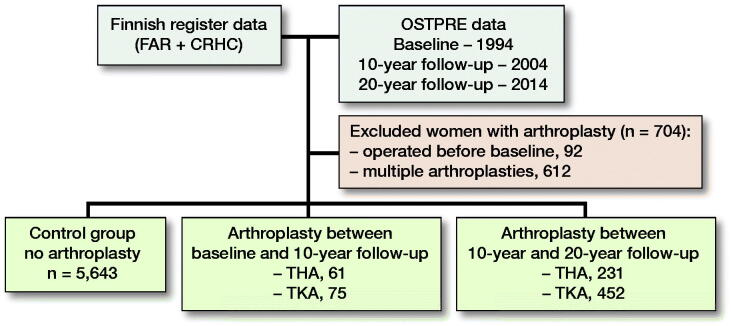
Flowchart of study population (N = 6,462).

The following subgroups of women were created (Figure 1, and see Tables 2 and 3): (1) the control group included all OSTPRE participants without arthroplasty until the end of follow-up; (2) women with hip or knee arthroplasty between baseline and 10-year follow-up; (3) women with hip or knee arthroplasty between 10-year and 20-year follow-up.

**Table 2.** Self-reported physical capability (PC) assessed by walking capability, in controls (women with no arthroplasty) and in women with total hip arthroplasty (THA) or total knee arthroplasty (TKA) at baseline, 10-year and 20-year follow-ups (%)

	n ^a^	Walking without limitations **^b^**	< 1 km **^c^**	< 100 m **^d^**	p **^e^**
Control group					
Baseline	5,356	95	4.0	1.0	
10-year follow-up	5,557	94	4.0	2.0	
20-year follow-up	5,497	80	11	9.0	
THA between baseline and 10-year follow-up					
Baseline	56	84	13	4.0	0.2
10-year follow-up	60	83	12	5.0	
20-year follow-up	59	64	12	24	
TKA between baseline and 10-year follow-up					
Baseline	73	84	15	1	0.01
10-year follow-up	73	80	16	4	
20-year follow-up	73	53	27	19	
THA between 10-year and 20-year follow-up					
Baseline	218	95	4	1	0.6
10-year follow-up	225	90	8	3	
20-year follow-up	222	76	12	12	
TKA between 10-year and 20-year follow-up					
Baseline	427	94	5	0	0.04
10-year follow-up	441	89	9	3	
20-year follow-up	431	71	17	12	

Arthroplasties are stratified by OSTPRE study follow-up periods (between baseline and 10-year follow-up, between 10-year and 20-year follow-up).

**^a^** Participants with valid answer in each individual follow-up point.

**^b^** “Good PC.”

**^c^** Can move < 1 km independently but > 100 m.

**^d^** Can move < 100 m independently.

**^e^** Chi-square was used to study the statistical significance of the changes in good PC during the follow-up between the control group and women with THA/TKA.

**Table 3.** Subjective well-being (SW) in controls (women with no arthroplasty) and in women with total hip arthroplasty (THA) or total knee (TKA) arthroplasty at baseline, 10-year, and 20-year follow-ups (%)

	n ^a^	Good	Moderate	Poor	p-value **^a^**
Control group					
Baseline	5,520	48	42	10	
10-year follow-up	5,593	52	45	3	
20-year follow-up	5,577	43	50	7	
THA between baseline and 10-year follow-up					
Baseline	60	33	50	17	0.01
10-year follow-up	60	33	62	5	
20-year follow-up	58	31	50	19	
TKA between baseline and 10-year follow-up					
Baseline	73	32	51	18	0.005
10-year follow-up	74	41	55	4	
20-year follow-up	74	27	57	16	
THA between 10-year and 20-year follow-up					
Baseline	227	45	46	9	0.004
10-year follow-up	227	38	56	5	
20-year follow-up	224	37	56	7	
TKA between 10-year and 20-year follow-up					
Baseline	435	40	49	11	< 0.001
10-year follow-up	445	36	60	4	
20-year follow-up	449	29	61	10	

Arthroplasties are stratified by OSTPRE-study follow-up periods ­(between baseline and 10-year follow-up, between 10-year and 20-year follow-up).

**^a^** Participants with valid answer in each individual follow-up point.

**^b^** Chi-square was used to study the statistical significance of the changes in good SW during the follow-up between the control group and women with THA/TKA.

### Statistics

We used the chi-square test to examine similarity of proportions of the population being in a certain physical capability state at different follow-up points between the control group and the different groups of women with THA/TKA. We used 1-way analysis of variance (ANOVA) to compare means of, e.g., height, weight, and BMI. We used propensity score matching to select the most suitable controls for women operated in with THA or TKA . The variables found in Characteristics (Table 1) were used as covariates. Statistical analysis was conducted with the Statistical Package for the Social Sciences (SPSS), version 27 (IBM Corp, Armonk, NY, USA).

**Table 1.** Characteristics of the study population (N = 6,462)

Factor	No arthroplasty during follow-up (n = 5,643)	Women with hip prostheses during follow-up (n = 292)	Women with knee prostheses during follow-up (n = 527)	p-value
Age at baseline (years)	57 (52–62)	57 (52–62)	57 (52–62)	< 0.001 **^a^**
Height (cm)	161 (136–179)	162 (147–176)	162 (143–178)	0.005 **^a^**
Weight (kg)	69 (38–125)	70 (47–103)	74 (48–120)	< 0.001 **^a^**
BMI	26 (16–53)	27 (19–40)	28 (20–48)	< 0.001 **^a^**
Mean number of chronic				
diseases at baseline	1.6 (0–10)	1.7 (0–8)	1.8 (0–9)	0.002 **^a^**
at end of follow-up	6.8 (0–36)	7.2 (0–26)	7.2 (0–26)	< 0.001 **^a^**
Self-reported diseases at end of follow-up (%) ^b^				
Osteoporosis/osteopenia	11	8.6	12	0.3
Rheumatoid arthritis/				
ankylosing spondylitis	4.1	6.2	8.3	< 0.001
Chronic back pain	24	30	29	0.004
Ischemic heart disease	18	16	19	0.7
Hypertension	58	59	66	0.002
Other heart disease	15	18	18	0.1
Asthma	14	15	14	0.9
Emphysema	2.6	2.7	2.5	1.0
Diabetes	17	14	22	0.006
Stroke	9.8	9.6	8.3	0.6
Cancer	14	13	12	0.6
Self-reported fractures (%) ^b^				
Hip fracture				
at baseline	0.1	0.0	0.0	0.8
end of follow-up	0.5	5.1	0.8	< 0.001
Any low trauma energy fracture				
at baseline	8.2	7.5	6.6	0.4
end of follow-up	12	14	13	0.4

**^a^** One-way analysis of variance (ANOVA).

**^b^** Pearson’s chi-square.

### Ethics, funding, and potential conflicts of interest

The Research Ethics Committee of the Northern Savo Hospital District has given permission for the OSTPRE study (3/11/2014//78/2004). Written consent has been provided by every study participant. The Finnish Institution for Health and Welfare has granted permission to use the CRHC and FAR data (THL/20/5.05.00/2016). This study was supported by the Finnish Arthroplasty Association, Päivikki and Sakari Sohlberg Foundation and Academy of Finland. The authors have no conflicting interests to report.

## Results

The overall study population consisted of 6,462 women, 292 of whom had THA and 527 whom had TKA. Hip fracture was the indication for THA in 9 women.

**Figure 2.**
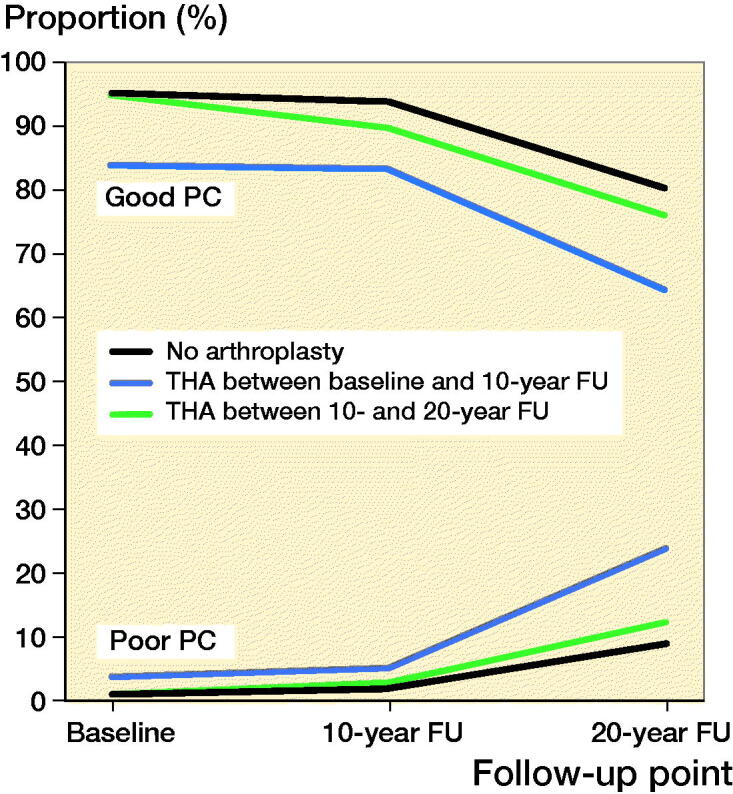
Proportion of women with good and poor self-reported physical capability (PC) after total hip arthroplasty (THA).

**Figure 3.**
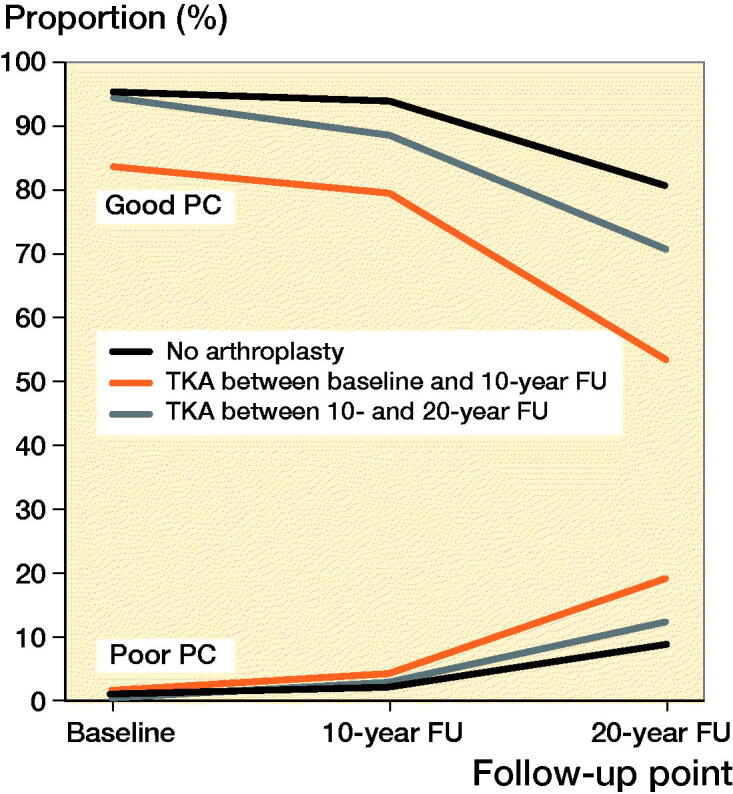
Proportion of women with good and poor self-reported physical capability (PC) after total knee arthroplasty (TKA).

**Figure 4.**
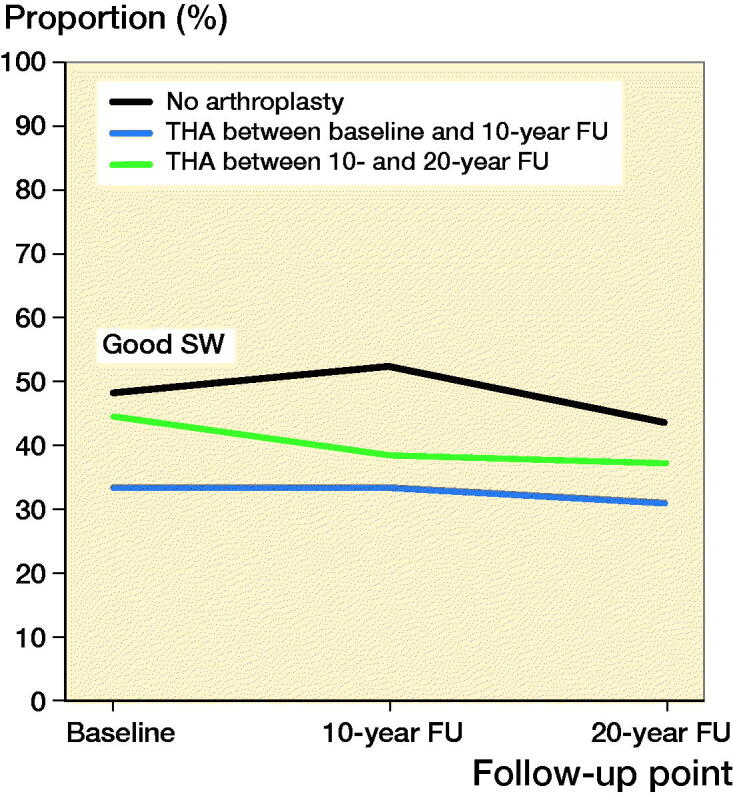
Proportion of women with good self-reported subjective wellbeing (SW) after total hip arthroplasty (THA).

**Figure 5.**
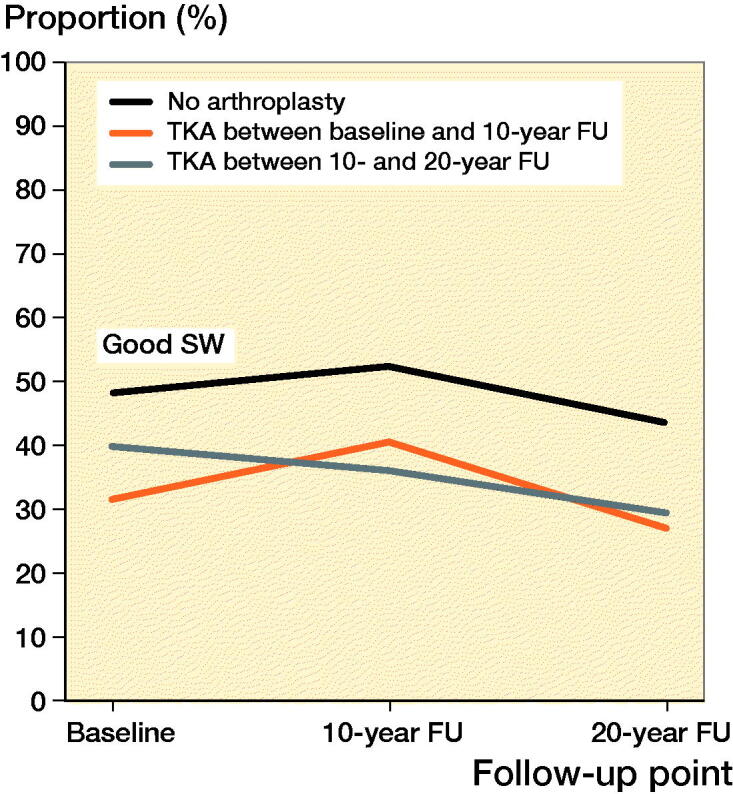
Proportion of women with good self-reported subjective wellbeing (SW) after total knee arthroplasty (TKA).

Among women with arthroplasty between baseline and 10-year follow-up, the mean age at the time of arthroplasty was 64 (THA)/65 (TKA) years. The median follow-up time for the groups was THA 13 (10–20)/TKA 12 (9–19) years. Good PC was reported by 83% of women with THA and 80% of women with TKA, at the 10-year follow-up (1st postoperative questionnaire). At the 20-year follow-up (2nd postoperative questionnaire), and good PC was reported by 64%/53% of women. The changes in good PC of women with a THA were not statistically significantly different from the control group (p = 0.2) whereas the changes in good PC in the TKA group were significantly different (p = 0.01) (Table 2 and Figures 2–3 and Table 5, see Supplementary data). Both THA and TKA women reported maintained or improved good SW after operation, at the 10-year follow-up 33% (THA)/41% (TKA). Later, at the 20-year follow-up, 31%/27% reported good SW. Again, there were statistically significant differences in good SW between THA (p = 0.01)/TKA (p = 0.005) and the control group (Table 3 and Figures 4–5, see Supplementary data). The proportion of women with revision arthroplasties until the end of follow-up was 21% for THA. Their results for good PC were: 92% (baseline), 77% (1st postoperative questionnaire) and 62% (2nd postoperative questionnaire). In this revised group, the proportion of women with good SW reports at the same follow-up points were 46%, 15%, and 18%. Only 6.7% of women with a TKA had revision arthroplasties.

Among women with THA or TKA between 10-year and 20-year follow-up, the mean age at the time of arthroplasty was 70 years for both THA and TKA. The median follow-up time for these women was THA 3 (0–9)/TKA 3 (0–10) years. Good PC was reported by 76% of women with THA and 71% with TKA at the 20-year follow-up (postoperative questionnaire) (Table 2 and Figures 2–3 and Table 5, see Supplementary data). The changes in good PC of women with THA were not significantly different (p = 0.6) when compared with the control group. For TKA there was a statistically significant difference (p = 0.04). During follow-up checkpoints participants reported a steady decrease of SW. Eventually, at the 20-year follow-up, good SW was reported by 37% (THA) and 29% (TKA) (Table 3 and Figures 4–5, see Supplementary data). Statistically the changes in proportion of women with good SW were significantly different from controls with THA (p = 0.004) and TKA (p < 0.001) women. Only 3.0% of the women with THA and 2.2% of women with TKA had experienced a revision arthroplasty by the end of follow-up.

Among OSTPRE participants without THA or TKA during follow-up, good PC was reported by 95% at baseline, 94% at 10-year follow-up, and 80% at 20-year follow-up (Table 2). SW remained almost the same throughout the follow-up (Table 3). At baseline 48% of these women reported good, 42% moderate, and 10% poor SW. The women in the control group and in the groups with a THA or TKA are similar in terms of age, height, number of low trauma energy fractures, osteoporosis/osteopenia, and the mean number of chronic diseases (Table 1). However, there are differences in the proportions of self-reports of some important groups of chronic diseases and in the amount of self-reported hip fractures by THA patients versus other participants.

Good PC was reported by 94–97% (baseline), 93–95% (10-year follow-up), and 80–81% (20-year follow-up) of the propensity score matched controls. Good SW was reported by 52–61% (baseline), 52–56% (10-year follow-up), and 47–53% (20-year follow-up) (Table 4).

**Table 4.** PC and SW results (%) for propensity score matched controls (no arthroplasty) for women with THA (n = 61) and TKA (n = 75) between baseline and 10-year follow-up, and for women with THA (n = 231) and TKA (n = 452) between 10-year and 20-year follow-up

		PC				SW		
	Walking without limitations **^a^**	< 1 km **^b^**	< 100 m **^c^**	p-value **^d^**	Good	Moderate	Poor	p-value ^d^
Baseline–10-year follow-up								
THA controls								
Baseline	95	6	0		61	31	9	
10-year follow-up	95	5	0	0.2	54	39	7	< 0.001
20-year follow-up	80	10	10		53	35	12	
TKA controls								
Baseline	94	6	0		59	32	9	
10-year follow-up	95	4	1	< 0.001	56	39	5	< 0.001
20-year follow-up	81	8	11		53	37	11	
10–20-year follow-up								
THA controls								
Baseline	97	3	0		54	38	8	
10-year follow-up	95	4	1	0.5	53	44	3	< 0.001
20-year follow-up	80	8	12		48	46	6	
TKA controls								
Baseline	97	3	1		52	40	9	
10-year follow-up	93	5	3	0.02	52	44	4	< 0.001
20-year follow-up	81	10	9		47	47	6	

**^a^** “Good PC.”

**^b^** Can move < 1 km independently.

**^b^** Can move < 100 m independently.

**^d^** Chi-square was used to study the statistical significance of the changes in good PC and SW through the follow-up between the propensity score matched controls and women with THA or TKA.

Analysis for women with THA or TKA within a 1-year period of any questionnaire showed that preoperatively 54% of THA and 65% of TKA participants reported good PC. Postoperatively good PC was reported by 62% of THA and 69% of TKA participants. Similarly, good SW was reported by 21% of THA and 24% of TKA participants preoperatively, but 37% (THA)/31% (TKA) postoperatively.

## Discussion

Elderly women who had experienced THA or TKA maintained their self-reported PC approximately 10 years after the procedure. However, at the end of follow-up the PC and SW among women with arthroplasty generally seemed to decrease more than in women without arthroplasty. 2 prior studies reported worse physical functioning 12 years after THA than in a control group without arthroplasty (Mariconda et al. 2011, Gould et al. 2012). Another study reported good yet deteriorating results from TKA patients 20 years after TKA (Meding et al. 2012). The women with arthroplasty between baseline and 10-year follow-up may have been affected more by osteoarthritis or other comorbidities before the operation than those who underwent the operation later, because before operation there were notably fewer women reporting good PC than in the control group. Postoperatively, the THA group seemed to benefit more from the operation. Previous reports on THA outperforming TKA support this finding (Ethgen et al. 2004). SW was improved or maintained with both THA/TKA at the first postoperative follow-up (10-year follow-up). However, at the 20-year follow-up, SW deteriorated and was a little worse than at baseline. The exact cause for deteriorating results during the longer follow-up (about 13 years postoperatively) remains unclear. Age and comorbidities related to aging may be the main factors, as at the 20-year follow-up the results decreased in all other groups too. Women with arthroplasty may be more prone to these factors. Women who had arthroplasty between baseline and 10-year follow-up were 5–6 years younger than those with arthroplasty later in life. Previous studies have reported that younger patients may be less satisfied with their THA or TKA operation. Regardless of good clinical results, they report more residual symptoms and their health-related quality of life may be more impaired than amongst older patients (Gotze et al. 2006, Parvizi et al. 2014). It may be that arthrosis worsens physical capability in an otherwise more physically capable young population, and arthroplasty restores capability later. Furthermore, changing social demands, i.e., during working life or doing sports without worrying about a prosthesis may have an influence on the improvement of PC. There are also patient-related factors that can influence postoperative patient-reported outcomes, e.g., comorbidities, obesity, psychological status, and expectations (Hofstede et al. 2016, Canovas and Dagneaux 2018).

Women who underwent THA/TKA later in life (between 10-year and 20-year follow-up) seemed to have a quite similar PC to the control group, 10 years prior to arthroplasty, at baseline. Before arthroplasty, at the 10-year follow-up, there was slight decrease in PC results, probably due to progression of osteoarthritis of the index joint. The postoperative scores in PC were close to those reported by the control group, and age-related factors may decrease patients’ physical capabilities even more than arthrosis. However, neither THA nor TKA completely restored a person’s ability to walk. The SW of these older women was good throughout the follow-up. Previous data has shown that age is not an obstacle for an effective THA or TKA and elderly people report improved quality of life scores after THA/TKA operations (March et al. 1999, Ethgen et al. 2004).

THAs’ and TKAs’ positive effects on pain, physical functioning and health are known to mostly increase from months to up to 2 years post-operatively (Ethgen et al. 2004, Williams et al. 2013). In our study, both PC and SW improved in participants with THA or TKA within 1 year of the questionnaire, when compared with results prior to operation.

At baseline, controls and women with arthroplasty had almost the same amount of doctor-diagnosed chronic diseases. At the end of follow-up, women with arthroplasty had a slightly higher average amount of chronic diseases. The greater burden of diseases may also affect PC and SW among women with THA or TKA. Furthermore, women with TKA had the highest average BMI as compared with controls and the THA group, which may have affected their PC negatively. Obesity has been shown to be strongly related to knee osteoarthritis but less to osteoarthritis of the hip (Hunter and Bierma-Zeinstra 2019).

We additionally performed propensity score matching, which gave PC results similar to the original control group. The difference in SW results between women with arthroplasty and propensity score matched controls was increased compared with the original control group.

Strengths of this study are the large cohort study combined with the national registers and with long-term data. Weaknesses of our study are that we did not have conclusive data on symptomatic joint diseases in the study population, and our results may not be generalizable to men. Also, no validated patient-reported outcome measures were used. However, scores used to evaluate clinical results of arthroplasty (e.g., Knee Society Score, Harris Hip Score, and Oxford Knee and Hip Score), include walking distance as a variable. Thus, our end point variable may be considered feasible for evaluation of the functional status. In addition, there are prior studies validating different self-reports in OSTPRE. Recently, we have reported the validity of self-reported physical capability with functional tests in the OSTPRE cohort (Juopperi et al. 2021). Also, self-reported fractures (Honkanen et al. 1999) as well as all hip fractures (Sund et al. 2014) have been validated. During follow-up there were many dropouts. The OSTPRE cohort is one of the rare true population-based cohorts of aging women with very long follow-up time and is also a part of the national roadmap infrastructure (Finnish Research Infrastructure for Population Based Surveys—FIRI-PBS). It is obvious that in the aging population there will be natural reasons for “dropout” in the population answering questionnaires, such as mortality or long-term institutionalization. In the OSTPRE cohort this has been compensated with record linkage to national registers. The PC or SW are not available in the registers, so without assuming some values for observable events (mortality, hospitalization, long-term institutionalization) we are forced to stick to the people who have answered the questionnaires. It is true that in this situation there may be some selection bias because of dropout. However, part of the dropout is not interesting at all, because we are interested in the population who can live a normal life with THA/TKA, not in those who have already died (17%) or ended up in an institution (10%), account for the 27% at the time of 20-year follow-up (i.e., at OSTPRE 25-year follow-up in 2014). Excluding women with these reasons from the dropout population makes the dropout rates much more tolerable. It is still possible and likely that the women who have answered are relatively healthier than the ones unwilling to participate anymore, but it is difficult to control for this kind of non-random bias.

In conclusion, THA and TKA maintain self-reported PC and SW. Yet, the overall PC and SW are lower in women with prior arthroplasty, in comparison with age-matched controls without arthroplasty. THA seems to outperform TKA in maintaining PC.

## Supplementary Material

Supplemental Material

## Supplementary data

Figures 2–5 and Table 5 are available as supplementary data in the online version of this article, http://dx.doi.org/10.1080/17453674.2021.1922039
