# Supplementary material for: Physical capability after total joint arthroplasty: long-term population-based follow-up study of 6,462 women
Source: Acta Orthop. 2021 May 12;92(5):551–6. doi: 10.1080/17453674.2021.1922039 (PMC8519522; doi:10.1080/17453674.2021.1922039)
Supplement: Supplemental Material [file IORT_A_1922039_SM3738.pdf]

Supplementary data

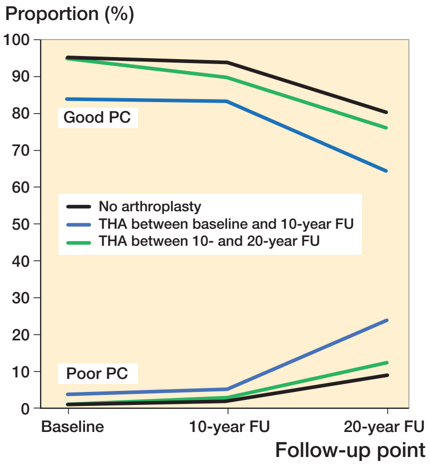

Figure 2. Proportion of women with good and poor self-reported physical capability (PC) after total hip arthroplasty (THA).

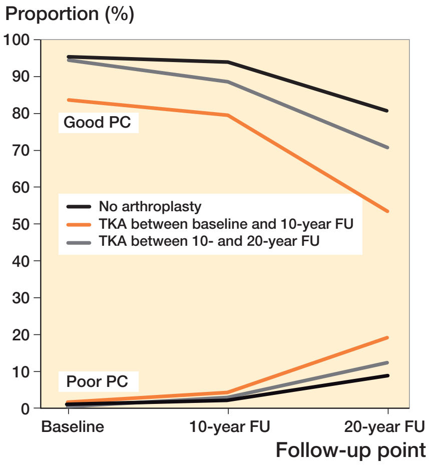

Figure 3. Proportion of women with good and poor self-reported physical capability (PC) after total knee arthroplasty (TKA).

Table 5. Proportion (%) of women reporting good physical capacity (PC) and good subjective well-being (SW) after THA and TKA stratified by age at the time of arthroplasty

Mean age	Proportion (%) < 4 [a]	< 13 [a]
Good PC after THA		
70	76	
64	83	64
Good PC after TKA		
70	71	
65	80	53
Good SW after THA		
70	37	
64	33	31
Good SW after TKA		
70	29	
65	41	27

[a] Postoperative time (years)

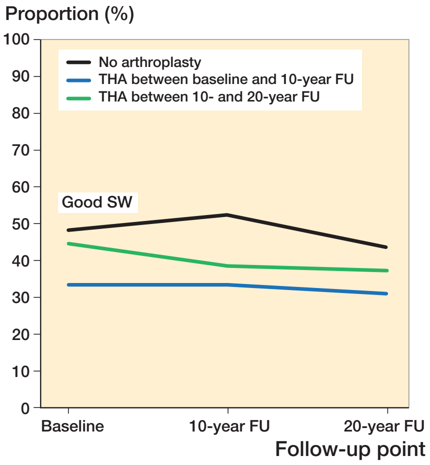

Figure 4. Proportion of women with good self-reported subjective wellbeing (SW) after total hip arthroplasty (THA).

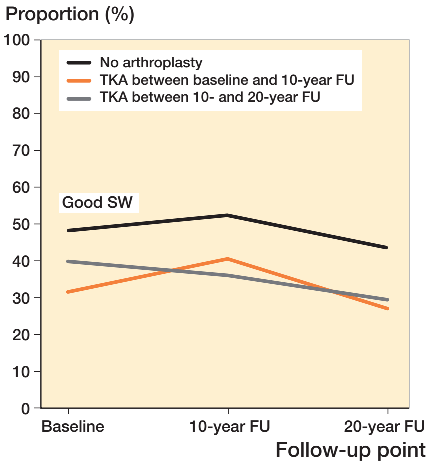

Figure 5. Proportion of women with good self-reported subjective wellbeing (SW) after total knee arthroplasty (TKA).
